# Semaphorin 3 C enhances putative cancer stemness and accelerates peritoneal dissemination in pancreatic cancer

**DOI:** 10.1186/s12935-023-03008-3

**Published:** 2023-08-03

**Authors:** Satoshi Tomizawa, Shigetsugu Takano, Ryotaro Eto, Tsukasa Takayashiki, Satoshi Kuboki, Masayuki Ohtsuka

**Affiliations:** Department of General Surgery, Graduate School of Medicine, Chiba University, Chiba, 260-8677 Japan

**Keywords:** SEMA3C, Pancreatic cancer, Peritoneal dissemination, Chemoresistance, Cancer stemness

## Abstract

**Purpose:**

Semaphorins, axon guidance cues in neuronal network formation, have been implicated in cancer progression. We previously identified semaphorin 3 C (SEMA3C) as a secreted protein overexpressed in pancreatic ductal adenocarcinoma (PDAC). We, therefore, hypothesized that SEMA3C supports PDAC progression. In this study, we aimed to investigate the clinical features of SEMA3C, especially its association with chemo-resistance and peritoneal dissemination.

**Methods:**

In resected PDAC tissues, we assessed the relationship between SEMA3C expression and clinicopathological features by immunohistochemistry. In vitro studies, we have shown invasion assay, pancreatosphere formation assay, colony formation assay, cytotoxicity assay, and activation of SEMA3C downstream targets (c-Met, Akt, mTOR). In vivo, we performed a preclinical trial to confirm the efficacy of SEMA3C shRNA knockdown and Gemcitabine and nab-Paclitaxel (GnP) in an orthotopic transplantation mouse model and in peritoneal dissemination mouse model.

**Results:**

In resected PDAC tissues, SEMA3C expression correlated with invasion and peritoneal dissemination after surgery. SEMA3C promoted cell invasion, self-renewal, and colony formation in vitro. We further demonstrated that SEMA3C knockdown increased Gem-induced cytotoxicity by suppressing the activation of the Akt/mTOR pathway via the c-Met receptor. Combination therapy with SEMA3C knockdown and GnP reduced tumor growth and peritoneal dissemination.

**Conclusions:**

SEMA3C enhances peritoneal dissemination by regulating putative cancer stemness and Gem resistance and activates phosphorylation of the Akt/mTOR pathway via c-Met. Our findings provide a new avenue for therapeutic strategies in regulating peritoneal dissemination during PDAC progression.

**Supplementary Information:**

The online version contains supplementary material available at 10.1186/s12935-023-03008-3.

## Backgrounds

Pancreatic ductal adenocarcinoma (PDAC) demonstrates a dismal outcome, with a five-year survival rate of approximately 10% [1]. PDAC recurrence is substantially high, with over 80% of cases recurring within two years, even after curative resection, owing to its high invasiveness and drug resistance [2, 3].

The major forms of recurrence are local recurrence, liver, lung, and peritoneal dissemination [3]. Peritoneal dissemination is established by several steps: detachment of cancer cells from a primary tumor, survival in the free abdominal cavity, attachment to the distant peritoneum, invasion into the subperitoneal space, and colonization [4, 5]. To achieve this, the capacity for invasiveness and cancer stemness is likely required; however, the underlying molecular mechanism of peritoneal dissemination remains unclear.

In the tumor microenvironment (TME), autocrine or paracrine secretion of proteins from cancer cells play crucial roles in cancer progression. Semaphorins (SEMAs) are a large family of axon guidance cues involved in neuronal network formation and are implicated in the progression of several malignancies [6, 7]. We previously identified semaphorin 3 C (SEMA3C) as a secreted autocrine protein whose expression is upregulated in murine PDAC cells compared with that in pre-cancerous pancreatic intraepithelial neoplasia (PanIN) cells by comprehensive secretome analysis [8].

In this study, we aimed to investigate the clinical features of SEMA3C and their correlation with peritoneal dissemination in resected PDAC tissues. We hypothesized that SEMA3C could exhibit the functional contributions of peritoneal dissemination with cancer stem cell (CSC)-like properties in PDAC. Furthermore, we conducted a preclinical study to investigate the potential of SEMA3C as a novel therapeutic target. Our findings would provide novel mechanistic insights into peritoneal dissemination via SEMA3C regulation and highlight its potential as a novel therapeutic target for PDAC.

## Materials and methods

### Patient samples and ethical considerations

Human PDAC tissues were obtained from 122 consecutive patients diagnosed with resectable PDAC between January 2013 and December 2017. Another cohort (112 locally advanced PDAC patients diagnosed between January 2008 and December 2019) was targeted to evaluate chemotherapy modification of PDAC tissues before surgery. All patients underwent curative pancreatectomy at the Department of General Surgery, Chiba University Hospital, and were histologically diagnosed with primary invasive PDAC. The 8th edition of the UICC was used to determine the TNM classification. Primary tumor volumes were measured from the resected specimen and calculated using the formula π/6 × (L ×W× W), where L and W are the longest and shortest tumor dimensions, respectively. Our protocol was approved by the Ethics Committee of Chiba University (protocol #2958), and written informed consent was obtained from each patient before the operation.

### Reagents and cell cultures

Human pancreatic duct epithelial (HPDE) cells and eight human pancreatic cancer cell lines (BxPC-3, MIA PaCa-2, PANC-1, Capan-2, AsPC-1, Hs766T, CFPAC-1, and Capan-1) were obtained from the American Tissue Culture Collection. Murine PDAC cell lines (PKCY cells) derived from a genetically engineered PKCY mouse (*Pdx1-cre; LSL-Kras*^*G12D/+;p53fl/+*^;*R26*^*YFP*^ mouse) were provided by Dr. Andrew D. Rhim (University of Texas, MD Anderson Cancer Center). Medium information for each cell line is listed in Table S1.

### Immunohistochemical staining

Immunohistochemistry was performed using formalin-embedded tissue blocks cut into 4-µm thick sections. Antigen retrieval was performed by autoclaving, and endogenous peroxidase activity was assessed using hydrogen peroxidase diluted to 3% with methanol. The sections were blocked with 5% bovine serum albumin (BSA) and incubated overnight at 4 ℃ with a primary antibody. Subsequently, the sections were incubated with a secondary antibody for 30 min at room temperature and visualized using diaminobenzidine (Nacalai Tesque). Antibody information is listed in Table S2. Staining patterns of SEMA3C were scored by two independent investigators using the histological score (H-score) (the product of the actual percentage of positively stained tumor cytoplasm and intensity score, evaluated as strong 3, moderate 2, and weak 1, yielding a possible range of 0–300).

### RNAi transfection and vector construct

SEMA3C siRNAs (Origene, Rockville, MD, USA) and control siRNA (AllStar negative control siRNA, QIAGEN, Hilden, Germany) were transfected into BxPC-3 cells at a concentration of 5 nmol/L using Lipofectamine™ RNAiMAX Reagent (Invitrogen, Carlsbad, CA, USA). SEMA3C was overexpressed in MIA PaCa-2 cells using Lipofectamine 3000 reagent (Invitrogen) by transfecting with a Human SEMA3C ORF clone (Genscript, Piscataway NJ USA), which was constructed using the pcDNA3.1+/C-(K)-DYK vector. The pcDNA3.1+/C-(K)-DYK blank vector was used as a negative control (GenScript). Mouse SEMA3C shRNA Lentiviral Transduction Particles (TRCN0000067389) and control shRNA (SHC016V-1EA) were purchased from Sigma-Aldrich (St. Louis, MO, USA). Following lentiviral infection, PKCY cells were selected using 5 µg/mL puromycin.

### RT-PCR

RNA was purified from the cell lines according to the manufacturer’s instructions using the RNeasy Mini Kit (Qiagen). Complementary DNA (cDNA) was synthesized from the mRNA using the SuperScript VILO cDNA Synthesis Kit and Master Mix (Invitrogen). Gene expression was quantified using the SYBR Green method with TB Green® FAST qPCR Mix (TaKaRa Bio Inc., Shiga, Japan). Primer information is listed in Table S3.

### Western blot analysis

Purified proteins were loaded onto a 5–12.5% XV PANTERA Gel (DRC, Tama, Tokyo, Japan) and transferred onto a polyvinylidene difluoride (PVDF) membrane. Non-specific proteins were blocked using 3% skim milk or Blocking One-P (Nacalai Tesque) for 60 min. The membranes were incubated with primary antibodies overnight at 4 °C and then incubated with secondary antibodies for 60 min at room temperature. Antibody information is listed in Table S2. The membranes were exposed to a chemiluminescent substrate (Nacalai Tesque) and analyzed using an LAS-4000UV image analyzer (Fujifilm, Tokyo, Japan). The intensity of each band was quantified by densitometry using ImageJ software and was used to calculate the relative protein levels normalized to β-actin or GAPDH.

### Invasion assay

BxPC-3 and MIA PaCa-2 cells were transfected with siRNA or pcDNA 24 h before the assay and seeded in the top chamber of a BioCoat Matrigel Invasion Chamber (Corning, New York, USA) at a density of 5 × 10^4^ cells per well in 400 µL non-FBS medium. The bottom chamber was filled with 500 µL medium containing FBS. Following incubation for 24 h, invaded cells on the bottom of the membrane were stained and counted in three randomly captured images.

### Flow cytometry

Pancreatic cancer stemness markers were analyzed using flow cytometry. Cells (1 × 10^6^) were centrifuged and simultaneously double stained to identify the CD44 and CD24 double-positive cell populations. CD133 and c-Met were used for single staining. The cells were suspended in primary antibodies for 60 min on ice in the dark. The data were compensated for spectral overlap and monitored using a FACS Canto II flow cytometer (Becton Dickinson, Franklin Lakes, NJ, USA). Expression levels were determined at 96 h after SEMA3C siRNA and pcDNA transfection. All data were analyzed using FlowJo v14.2.0 software (Ashland, OR, USA).

### Pancreatosphere formation assay

Pancreatosphere formation assays were executed as previously described [9]. Briefly, Cells were seeded in 96-well ultra-low attachment plates (Corning) at a density of 10 cells per well and incubated for seven days in sphere medium. To assess the sphere formation rate, we counted the viable cells based on trypan blue exclusion and divided the total number of plated cells.

### Colony formation assay

Colony formation assays were executed as previously described [10]. Briefly, BxPC-3 or MIA PaCa-2 cells were seeded in 24-well plates at a density of 3000 cells per well. Colonies in each well were counted two weeks after seeding.

### Lactate dehydrogenase cytotoxicity assay

The cytotoxicity of gemcitabine (Gem) was determined by measuring lactate dehydrogenase (LDH) activity released from dead cells using the LDH Cytotoxicity Assay Kit (Nacalai Tesque) following the manufacturer’s protocol. At 120 h, the LDH released into the supernatant was measured after incubating the cells in 96-well plates in a medium with Gem. The percentage of cytotoxicity was calculated using the following formula:

% cytotoxicity = (experimental released LDH – low control) / (high control – low control) × 100,

where low and high controls denote spontaneous and total LDH released from the untreated and lysis solution-treated cells, respectively.

### In vivo orthotopic transplantation and peritoneal dissemination model

In the orthotopic transplantation model, 8–10-week-old male BALB/AJcl-nu/nu mice (CLEA Japan, Tokyo, Japan) were injected with 5 × 10^4^ PKCY cells suspended in 25 µL DMEM into the subcapsular region of the pancreatic tail under anesthesia. The mice were randomized to receive no treatment or gemcitabine plus nab-paclitaxel (GnP) on day 10. The GnP group received intraperitoneal (IP) injections at a dose of 120 mg/kg per week. On day 27 after transplantation, the mice were euthanized, and primary tumor volumes were calculated.

In the peritoneal dissemination model, 1 × 10^5^ PKCY cells in 250 µL DMEM were injected into the peritoneum of the mice. The mice were randomized to “no treatment” or “GnP therapy” on day 7, and the GnP group received IP injections at a dose of 120 mg/kg per week. On day 21, after implantation, the mice were euthanized. Peritoneal dissemination was assessed by calculating the peritoneal carcinomatosis index (PCI). The peritoneal cavity was evaluated and divided into 13 zones, and tumor nodules and PCI scores were calculated as follows: 0, macroscopic tumor; 1, limited tumor growth (1–2 mm diameter); 2, moderate tumor growth (2–4 mm diameter); and 3, abundant tumor nodules (> 4 m diameter or five deposits).

### Statistical analysis

The significance of the difference in survival rates was analyzed by the log-rank test using the Kaplan–Meier method. Data are expressed as the mean ± SD. Cox proportional hazard models were used for univariate and multivariate survival analyses. Statistically significant differences were determined using Student’s t-test, Chi-square test, or Mann–Whitney–Wilcoxon test. Statistical significance was set at p < 0.05. Each experiment was replicated at least three times.

## Results

### High SEMA3C expression is correlated with the relapse of peritoneal dissemination

**Fig. 1:**
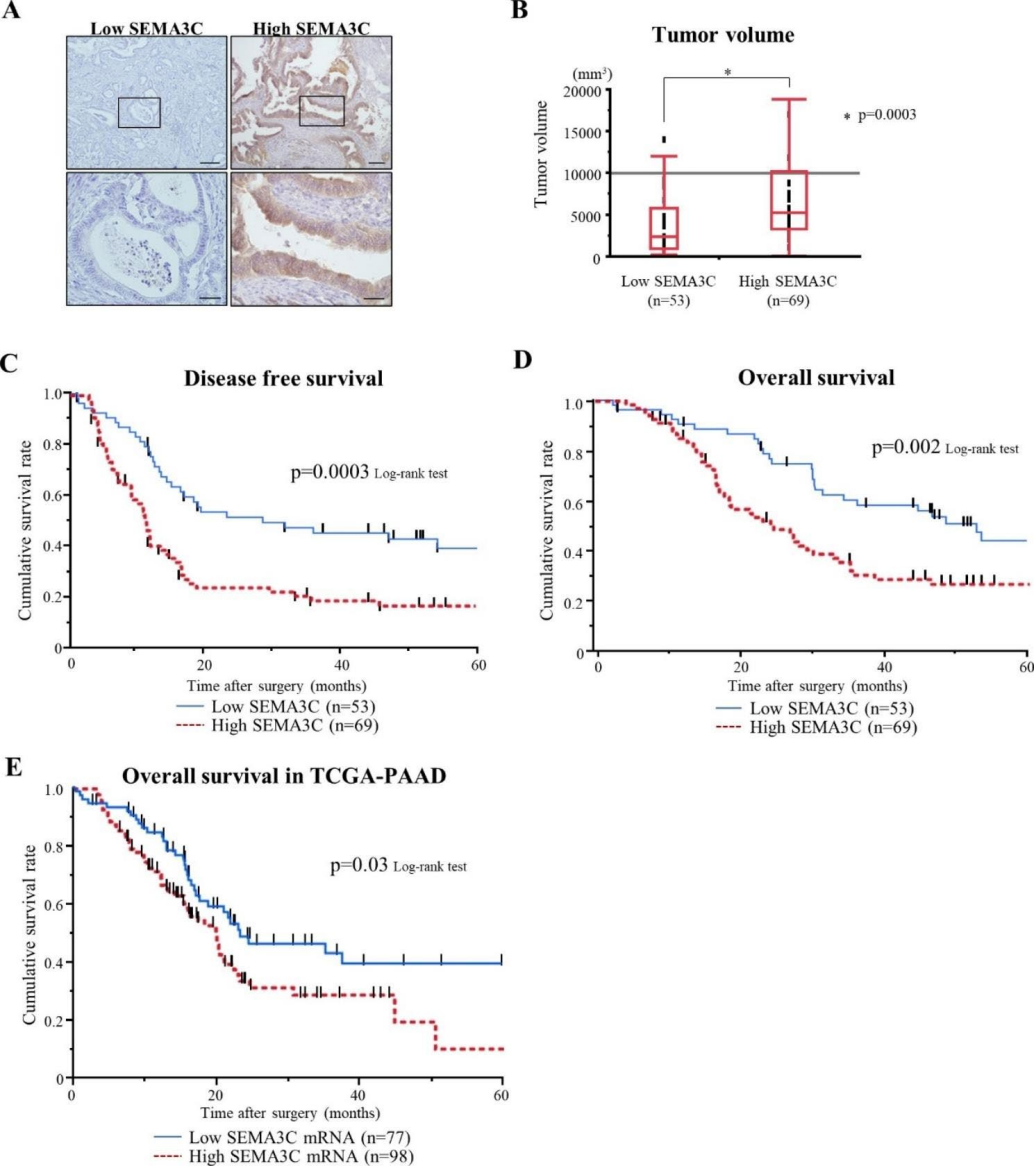
IHC analysis of SEMA3C expression in resected human PDAC samples. (**A**) Representative immunohistochemistry staining for SEMA3C in PDAC tissues. Original magnification: upper panels 100× (scale bars, 200 μm), lower panel 400× (scale bars, 50 μm). PDAC tissues were categorized into low (left panel) and high SEMA3C expression (right panel) based on the H-score. (**B**) Pancreatic tumor volumes are represented as box-plot histograms. Each volume represents the mean ± SEM. Kaplan–Meier analyses of PDAC patients with low vs. high SEMA3C expression for disease-free survival (low SEMA3C group; MST 28.7 months, high SEMA3C group; MST 11.3 months, p = 0.0003) (**C**) and overall survival (low SEMA3C group; MST 53.0 months, high SEMA3C group; MST 24.6 months, p = 0.002) (**D**). (**E**) Kaplan–Meier analyses of PDAC patients with low vs. high SEMA3C mRNA for overall survival in the TCGA PAAD dataset (low SEMA3C mRNA group; MST 23.4 months, high SEMA3C mRNA group; MST 119.8 months, p = 0.03)

We assessed the relationship between SEMA3C expression and clinicopathological features in patients with 122 resectable PDAC who had undergone upfront surgery. All tissue samples were scored by H-score and divided into two groups; 69 (56.6%) and 53 cases (43.4%) were classified as High and Low SEMA3C groups, respectively (Fig. 1A). The relationship between SEMA3C expression and clinicopathological findings in PDAC tissues is presented in Table 1. SEMA3C levels positively correlated with tumor volume (p = 0.0003) (Fig. 1B), and High SEMA3C levels were also associated with invasive features, such as lymphatic and neural invasion, compared with that in Low SEMA3C levels in primary PDAC. Notably, High SEMA3C levels had a significantly higher incidence of peritoneal dissemination after curative resection (p = 0.03) (Table 2). Kaplan–Meier analyses indicated that High SEMA3C levels had significantly shorter disease-free survival (p = 0.0003) (Fig. 1C) and overall survival (p = 0.002) (Fig. 1D) than those with Low SEMA3C levels. To validate these clinical data in an independent cohort, we evaluated SEMA3C mRNA expression in a publicly available pancreatic ductal adenocarcinoma dataset of The Cancer Genome Atlas (TCGA-PAAD) [11]. The patients of the TCGA-PAAD cohort were divided into two groups based on the same percentage as in our cohort, (High SEMA3C mRNA (98/175: 56%) and Low SEMA3C mRNA (77/175: 44%)). Similar to the above results, analysis of TCGA-PAAD dataset revealed that the High SEMA3C mRNA group again had a significantly worse prognosis compared to the Low SEMA3C mRNA group (p = 0.03) (Fig. 1E). Furthermore, High SEMA3C levels were identified as an independent prognostic factor in the multivariate analysis using the Cox proportional hazard ratio model (p = 0.008) (Table 3). These data suggest that High SEMA3C levels present an aggressive feature, leading to poor outcomes in PDAC patients with peritoneal dissemination.

**Table 1:** Relationship between SEMA3C expression and clinicopathological features of PDAC patients

Parameters	SEMA3C expression		P value
	High (n = 69)	Low (n = 53)	
Gender (Male/Female)	31/38	29/24	0.28
Age (y.o. median: range)	70 (38–82)	71 (50–86)	0.86
CA19-9 (median: range)	213.2 (0-15600)	105 (0.1–1787)	0.02
Tumor volume (mm^3^ median: range)	5233 (67-24647)	2355 (157-32153)	0.0003
UICC Stage (IA,IB,IIA,IIB/III,IV) ^a^	45/24	38/15	0.45
pT1, 2/pT3, 4 ^a^	2/67	8/45	0.01
pN0/pN1, 2 ^a^	20/49	16/37	0.89
ly (+/−)	68/1	44/7	0.006
v (+/−)	60/9	40/12	0.15
ne (+/−)	67/2	43/9	0.006
PL (+/−)	18/46	8/43	0.11

chi-square test, Mann–Whitney–Wilcoxon test

p; pathological findings, T; primary tumor, N; regional lymph nodes, ly; lymphatic invasion, v; venous invasion, ne; neural invasion, PL; extra pancreatic nerve plexus invasion

^a^ The Union for International Cancer Control 8th edition

**Table 2:** Relationship between SEMA3C expression and recurrence pattern of PDAC patients

Parameters		SEMA3C expression		P value
		High (n = 69)	Low (n = 53)	
Local recurrence (+/−)		22/47	13/40	0.37
Lymph node metastasis (+/−)		11/58	4/49	0.15
Hematogenous dissemination (+/−)		24/45	14/39	0.32
	Liver metastasis (+/−)	19/50	9/44	0.17
Peritoneal dissemination (+/−)		8/61	1/52	0.03

chi-square test

**Fig. 2:**
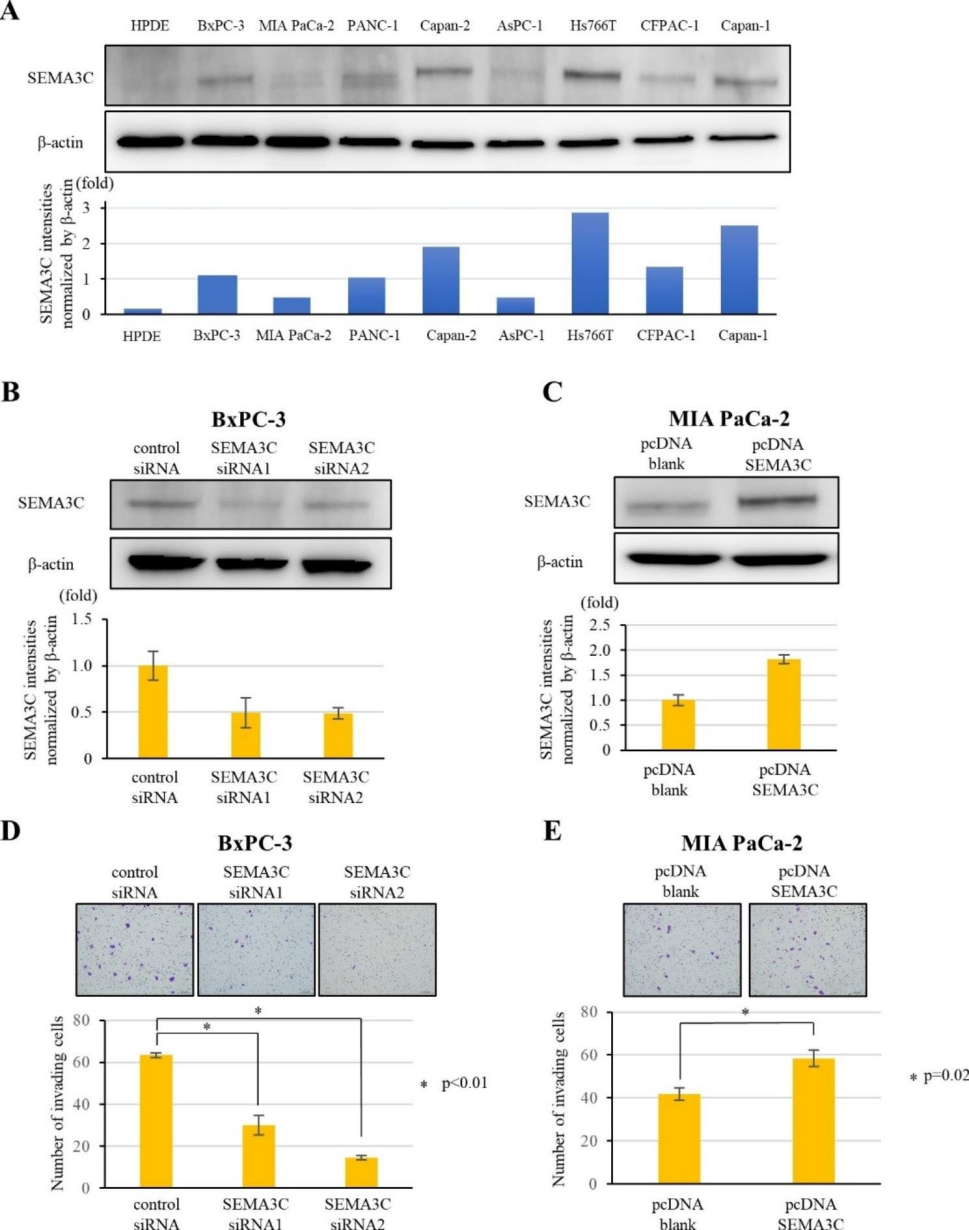
Differential expression of SEMA3C in different human PDAC cell lines. (**A**) Upper panels: SEMA3C protein expression in normal HPDE and human PDAC cell lines (BxPC-3, MIA PaCa-2, PANC-1, Capan-2, AsPC-1, Hs766T, CFPAC-1, and Capan-1) detected using western blot analysis. Lower panel: A comparative analysis of SEMA3C expression among cell lines. Effect of SEMA3C on cell invasion in PDAC cells. SEMA3C expression was evaluated by western blot analysis in BxPC-3 cells transfected control siRNA, SEMA3C siRNA1, and SEMA3C siRNA2 (**B**) and MIA PaCa-2 cells transfected pcDNA blank and pcDNA SEMA3C (**C**). Invasion assay comparing in BxPC-3 cells transfected with control siRNA, SEMA3C siRNA1, and SEMA3C siRNA2 (**D**), and MIA PaCa-2 transfected with pcDNA blank and pcDNA SEMA3C (**E**)

**Table 3:** Univariate and multivariate analyses of prognostic factors of PDAC patients

Parameters		Univariate analysis		Multivariate analysis	
	N = 122	Hazard ratio(95%CI)	P value	Hazard ratio(95%CI)	P value
Age (y.o.) (>/≤70)	62/60	1.39 (0.97-2.00)	0.07		
Gender (male/female)	60/62	0.95 (0.66–1.36)	0.78		
Tumor volume (>/≤ 4238 mm)	62/60	2.57 (1.61–4.12)	< 0.0001	1.88 (1.13–3.12)	0.01
Histological grade (Poorly/others)	14/108	1.49 (0.85–2.62)	0.16		
pT3, 4/pT1, 2 ^a^	112/10	3.12 (0.98–9.95)	0.05		
pN1, 2/pN0 ^a^	86/36	1.79 (1.21–2.66)	0.004	2.25 (1.24–4.08)	0.008
ly (+/−)	112/8	2.22 (1.06–4.65)	0.04	1.03 (0.11–9.97)	0.98
v (+/−)	100/21	1.93 (1.16–3.21)	0.01	2.91 (1.04–8.13)	0.04
ne (+/−)	110/11	1.73 (0.93–3.23)	0.09		
SEMA3C expression (High/Low)	69/53	1.63 (1.13–2.34)	0.008	2.24 (1.23–4.08)	0.008

Cox’s proportional hazard model

p; pathological findings, T; primary tumor, N; regional lymph nodes, ly; lymphatic invasion, v; venous invasion, ne; neural invasion

^a^ The Union for International Cancer Control 8th edition

### SEMA3C facilitates cell invasion in PDAC cells

To monitor SEMA3C expression in PDAC cells, SEMA3C protein expression was evaluated using western blotting in human PDAC cell lines. SEMA3C expression was higher in PDAC cell lines than in normal HPDE cells (Fig. 2A). BxPC-3 and MIA PaCa-2 were established as cells with high- and low expression of SEMA3C for further in vitro experiments. To explore the clinical characteristics of SEMA3C expression, we analyzed the functional ability of SEMA3C in PDAC in vitro. To assess the functional roles of autocrine SEMA3C, endogenous SEMA3C expression was knocked down using SEMA3C-specific siRNAs in BxPC-3 cells (Fig. 2B and Fig. S1A), and SEMA3C was overexpressed in MIA PaCa-2 cells by transfection with SEMA3C pcDNA (Fig. 2C and Fig. S1B). SEMA3C knockdown decreased cell invasion in BxPC-3 cells (Fig. 2D), whereas it was increased in MIAPaCA-2 cells (Fig. 2E). These findings demonstrated that SEMA3C increases the cellular invasiveness of PDAC cells in vitro.

### SEMA3C promotes cancer stem cell properties in PDAC cells

**Fig. 3:**
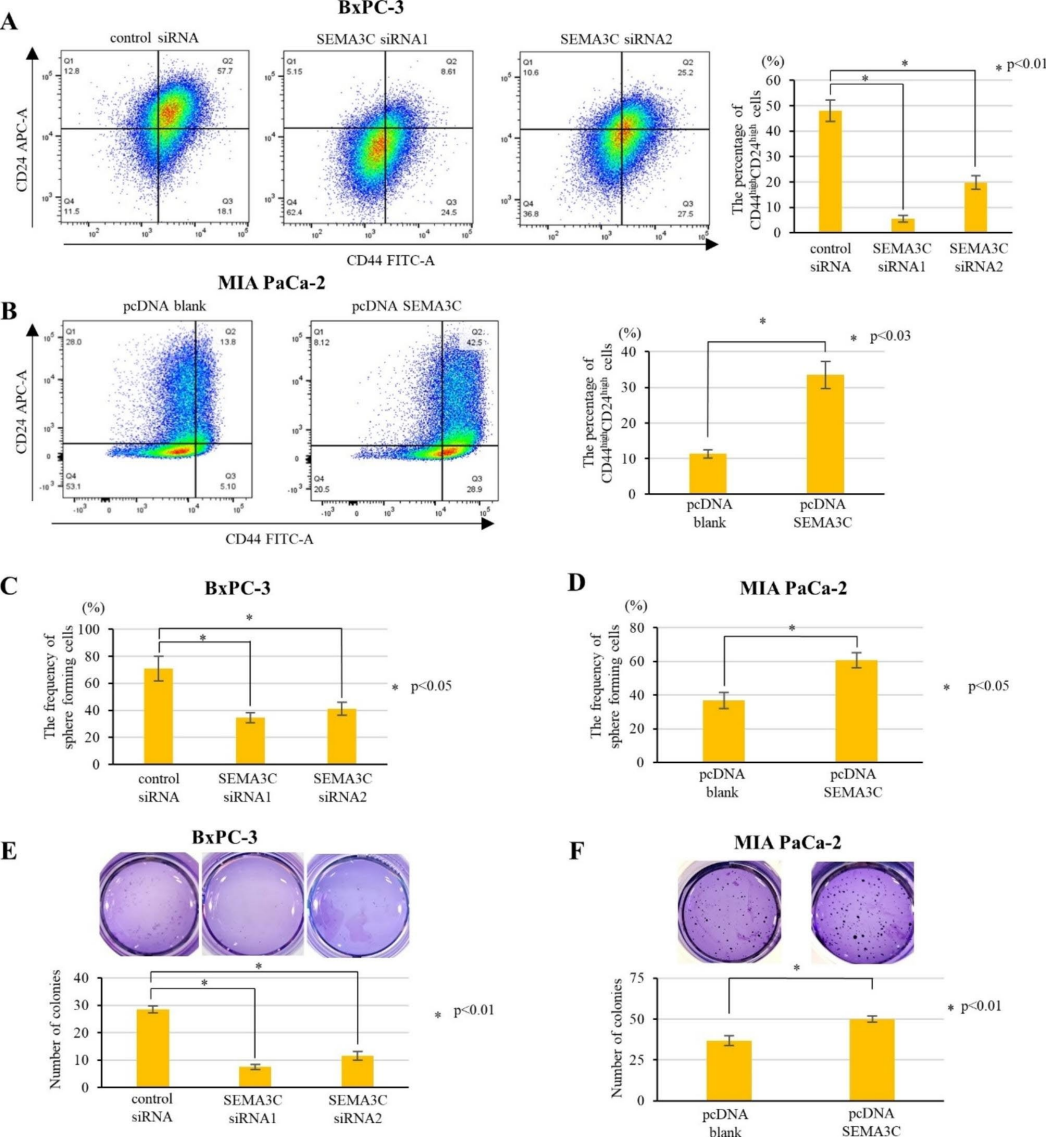
SEMA3C is associated with stem cell-like properties and promotes self-renewal and tumorigenicity in PDAC cells. (**A**) Left panels: Expression patterns of stem cell markers, such as CD44 and CD24, were compared in BxPC-3 cells transfected control siRNA, SEMA3C siRNA1, and SEMA3C siRNA2 by flow cytometry. Right panel: The percentage of CD44^high^24^high^ cells. (**B**) Left panels: Expression patterns of CD44 and CD24 in MIA PaCa-2 control and SEMA3C-overexpressing cells using flow cytometry. Right panel: The percentage of CD44^high^24^high^ cells. The sphere formation rate in BxPC-3 cells treated with control siRNA, SEMA3C siRNA1, and SEMA3C siRNA2 (**C**), and in MIA PaCa-2 cells control and SEMA3C-overexpressing cells (**D**). Colony formation assay with BxPC-3 cells treated with control siRNA, SEMA3C siRNA1, and SEMA3C siRNA2 (**E**), and MIA PaCa-2 control and SEMA3C-overexpression cells (**F**)

As putative CSC properties play a crucial role in cancer progression, we first analyzed the expression of the pancreatic CSC markers CD44, CD24, CD133, and c-Met [12–15]. Flow cytometry analyses revealed that the population of CD44^high^CD24^high^ was decreased in SEMA3C-knockdown BxPC-3 cells (Fig. 3A), whereas it increased with SEMA3C overexpression in MIAPaCa-2 cells (Fig. 3B) compared to with that in the respective control cells. The expression of CD133 and c-Met remained unaltered in SEMA3C knockdown or overexpression cells (Fig. S2A and B).

To assess the correlation between SEMA3C and self-renewal capacity, we performed a pancreatosphere formation assay in PDAC cells (10). Sphere-forming cells were significantly reduced in SEMA3C knockdown BxPC-3 cells (Fig. 3C) but increased in SEMA3C-overexpressing MIA PaCa-2 cells (Fig. 3D) compared with that in the respective control cells. Colony formation assay was performed to evaluate the ability of SEMA3C to colonize in vitro. Compared with that in the control cells, the capacity for colony formation was decreased in SEMA3C knockdown BxPC-3 cells (Fig. 3E), whereas it was increased in SEMA3C-overexpressing MIA PaCa-2 cells (Fig. 3F). These data highlight the potential role of SEMA3C in regulating CSC properties in PDAC in vitro.

### SEMA3C contributes to gemcitabine resistance via the activation of c-Met/AKT/mTOR signaling in PDAC cells

**Fig. 4:**
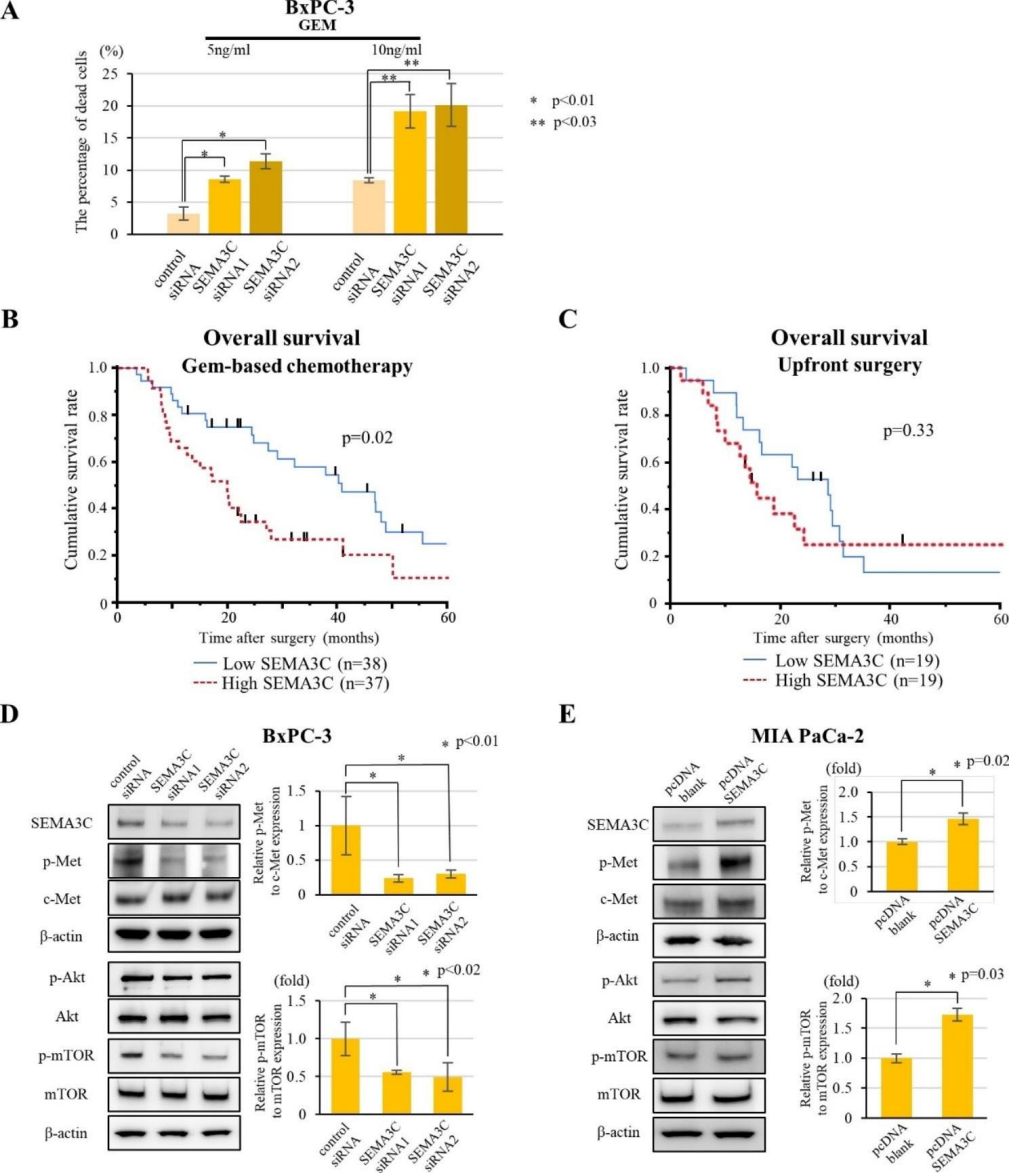
Correlation between SEMA3C and GEM-resistance in PDAC cells and patient samples. (**A**) LDH cytotoxicity assay of GEM comparing BxPC-3 cells transfected with control siRNA, SEMA3C siRNA1, and SEMA3C siRNA2. (**B**, **C**) IHC analysis of SEMA3C expression in locally advanced human PDAC samples. Kaplan–Meier analyses of locally advanced PDAC patients with high vs. low SEMA3C expression for overall survival in the NAC group (high SEMA3C group; MST 22.7 months, low SEMA3C group; MST 44.1 months, p = 0.02) (**B**), and in the upfront surgery group (high SEMA3C group; MST 15.5 months, low SEMA3C group; MST 28.7 months, p = 0.33) (**C**). The protein expression of c-Met, Akt, mTOR, and their phosphorylation using Western blot analysis in BxPC3 (**D**, left panel) and MIA-PaCa-2 cells (**E**, left panel). The expression of p-Met relative to c-Met and p-mTOR relative to mTOR in BxPC-3 cells (**D**, right panel) and MIA PaCa-2 cells (**E**, right panel)

Gem is widely used as a standard treatment for PDAC. CSCs are considered responsible for drug resistance, leading to cancer recurrence after surgery following chemotherapy. Therefore, we investigated the association between SEMA3C and Gem resistance. In vitro, we observed that SEMA3C knockdown resulted in a significant increase in the sensitivity to Gem at two different concentrations in BxPC-3 cells (Fig. 4A). Overexpression of SEMA3C decreased sensitivity to gemcitabine, but not significantly in MIA PaCa-2 cells (Fig. S3). Among the 112 resected locally advanced PDAC samples, 74 patients received Gem-based neoadjuvant chemotherapy (NAC; Gem and S-1 (GS) and GnP) followed by curative surgery, and 38 patients underwent upfront surgery. Kaplan–Meier analyses indicated that High SEMA3C was correlated with poor prognosis in the Gem-based NAC group, whereas no correlation was observed between SEMA3C and survival in the non-NAC upfront surgery group (Fig. 4B and C). These data imply that SEMA3C expression is correlated with Gem resistance in PDAC.

To elucidate the mechanism of Gem resistance in PDAC cells, we investigated the SEMA3C-related signaling pathway using western blotting. Phosphorylated-Met (p-Met), an activated form of c-Met, which is a receptor for SEMA3C, was decreased in SEMA3C knockdown BxPC-3 cells, whereas it was increased in SEMA3C overexpressing MIA PaCa-2 cells (Fig. 4D and E). Notably, the Akt/mTOR pathway, a downstream signaling pathway of the c-Met receptor, was also suppressed in SEMA3C knockdown BxPC-3 cells, whereas it was activated in SEMA3C overexpressing MIA PaCa-2 cells (Fig. 4D and E). These data suggest that SEMA3C activates the c-Met/Akt/mTOR signaling pathway in an autocrine fashion.

### SEMA3C knockdown suppresses tumor growth and enhances the antitumor effect of gemcitabine in an orthotopic transplantation mouse model

**Fig. 5:**
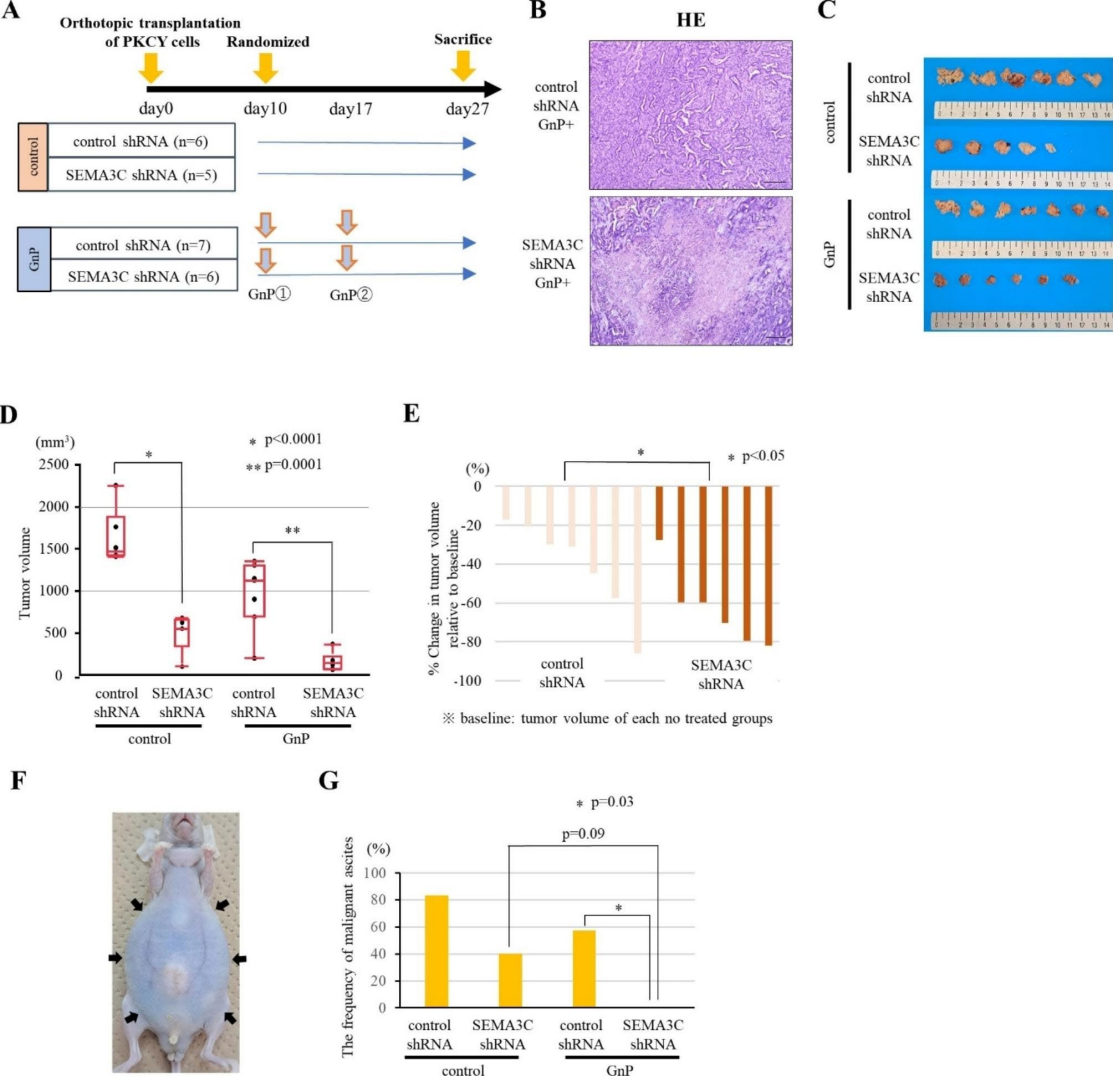
Preclinical trial to confirm GnP efficacy using the orthotopic transplantation mouse model. (**A**) The experimental design of the randomized trial (untreated group / GnP group; gemcitabine (GEM, 120 mg/kg) and nab-paclitaxel (nab-PTX, 120 mg/kg)) in the orthotopic transplantation model injected with control- and SEMA3C shRNA-transduced PKCY cells. (**B**) Paraffin-embedded orthotopic tumor tissues were sectioned and stained with H&E. (**C**) The indicated excised tumor of the orthotopic model. (**D**) Pancreatic tumor volumes are represented as box-plot histograms. Each volume represents the mean ± SEM. (**E**) Waterfall plot indicating changes in the volume compared with the baseline (mean tumor volume of each no treated group). (**F**) Image of a mouse with malignant ascites just before euthanasia. (**G**) The frequency of malignant ascites in mice just before sacrifice

To examine whether SEMA3C knockdown impairs tumor progression and enhances the sensitivity of Gem to PDAC in vivo, we performed a preclinical trial to confirm the efficacy of GnP using an orthotopic transplantation mouse model. PKCY cells transduced with control or SEMA3C shRNA was confirmed SEMA3C knockdown in western blot analysis (Fig. S4A). Sphere-forming cells were significantly reduced in SEMA3C knockdown PKCY cells (Fig. S4B) in vitro. PKCY cells were injected into the subcapsular region of the pancreatic tail of nude mice (Fig. S4C). After randomization, experimental mice were treated with GnP, and pancreatic tumors were harvested on day 27 after PKCY cell injection (Fig. 5A). In the GnP-treated group, representative microscopic findings revealed increased fibrotic denaturation with necrosis in the SEMA3C knockdown group compared with that in the control group (Fig. 5B). Tumor growth was significantly suppressed by SEMA3C knockdown in the untreated and GnP-treated groups (Fig. 5C and D). Specifically, the tumor reduction effect of the combination of GnP and SEMA3C knockdown was evaluated by measuring the change in tumor volume using the mean tumor volume of each untreated group as the baseline. The waterfall plot revealed increased tumor regression in the SEMA3C knockdown group than in the control group in the preclinical study (Fig. 5E). Focusing on malignant ascites caused by peritonitis carcinomatosa (Fig. 5F), no incidence was observed in only the SEMA3C knockdown group treated with GnP (Fig. 5G). These results suggest that SEMA3C knockdown suppresses tumor growth and enhances the antitumor effect of GnP in the orthotopic transplantation mouse model.

### SEMA3C knockdown with GnP therapy impairs peritoneal dissemination in vivo

**Fig. 6:**
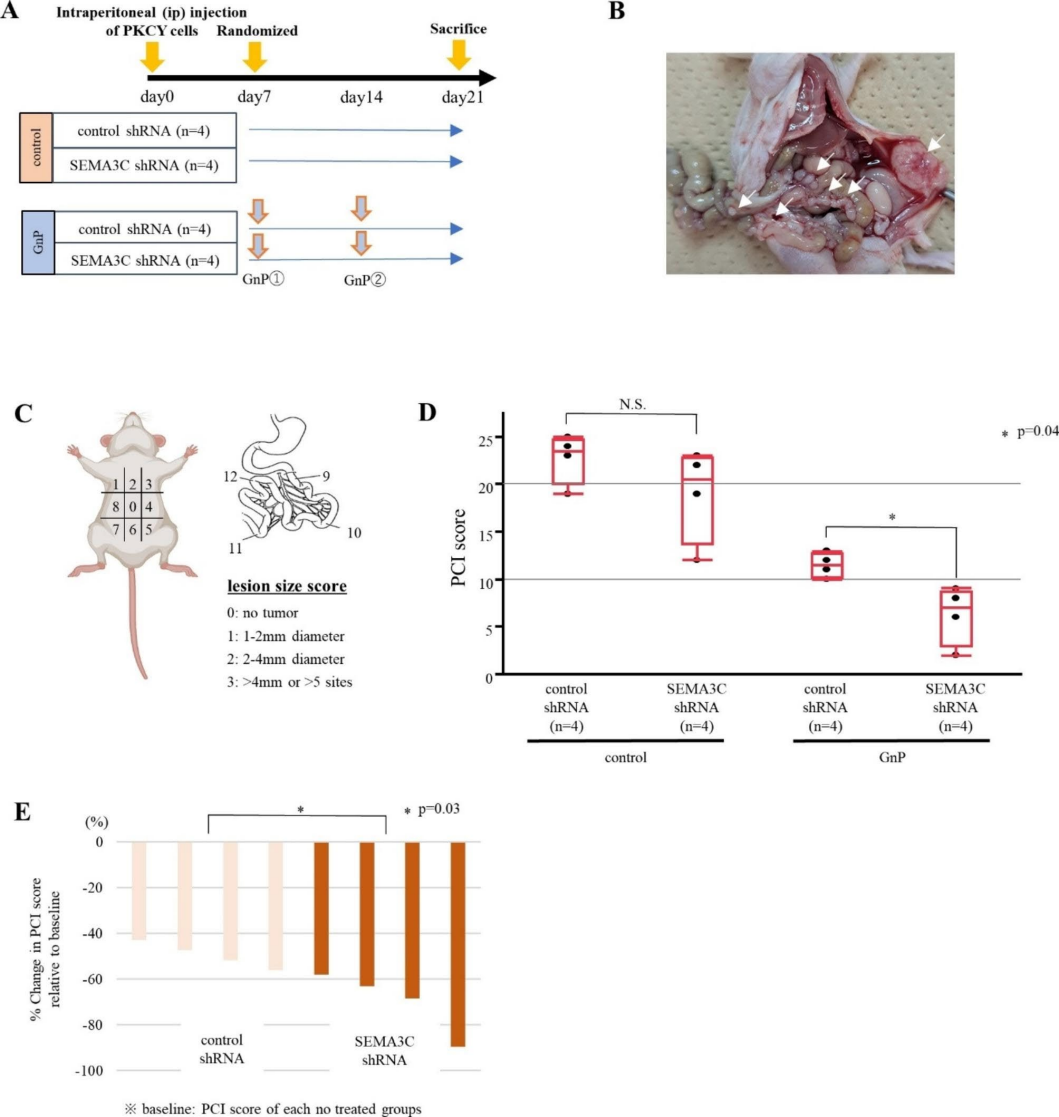
Preclinical trial using the peritoneal dissemination mouse model. (**A**) Experimental design of the peritoneal dissemination model (untreated group / GnP group; GEM- (120 mg/kg) and nab-paclitaxel (120 mg/kg))-injected PKCY cells transduced with control and SEMA3C shRNA into the peritoneal cavity. (**B**) Image depicting the distribution of peritoneal dissemination nodes localized majorly in the parietal peritoneum and mesentery. (**C**) Schematic overview of the PCI score to evaluate peritoneal dissemination in mice. (**D**) PCI score for the GnP group in box-plot histograms. Each score represents the mean ± SEM. (**E**) Waterfall plot indicating changes in the PCI score compared with baseline (mean PCI score of each no treated group)

Considering the IHC results demonstrating that SEMA3C was correlated with peritoneal dissemination in PDAC patients and promoted invasiveness and self-renewal capacity in vitro, we examined whether SEMA3C facilitates tumorigenesis and peritoneal colonization in vivo. PKCY cells transduced with control shRNA or SEMA3C shRNA were injected intraperitoneally. Experimental mice were treated with GnP and euthanized on day 21 after PKCY cell injection (Fig. 6A). The untreated control shRNA group exhibited significantly increased body weight with malignant ascites on day 21 compared with that in the other groups (Fig. S5, p = 0.01). Peritoneal nodules, mainly formed in the abdominal wall and mesentery, were analyzed using PCI scores (Fig. 6B and C). In the GnP-treated group, the PCI score exhibited a significant decrease between the control and SEMA3C shRNA groups (p = 0.04), whereas no significant difference was observed between the two groups in the untreated group (Fig. 6D). Notably, waterfall plot analysis indicated that the combination of GnP and SEMA3C knockdown significantly decreased peritoneal dissemination compared to control group (Fig. 6E). These data demonstrated that SEMA3C knockdown with Gem-based chemotherapy impaired peritoneal dissemination in vivo model.

## Discussion

**Fig. 7:**
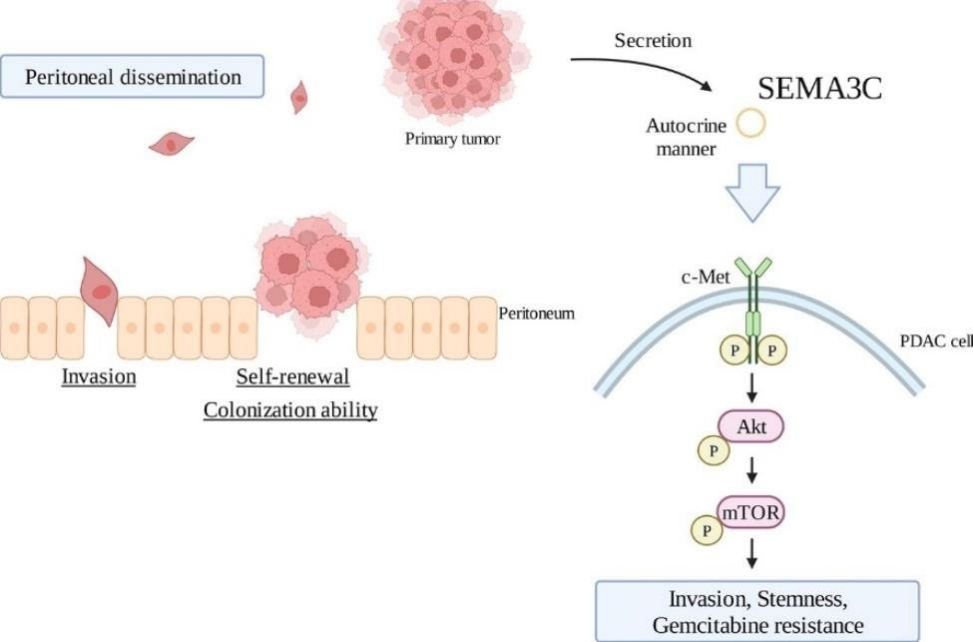
Schema of SEMA3C functions in peritoneal dissemination of PDAC

In this study, we demonstrated that SEMA3C, previously identified in the secretion of PDAC cells [8], is correlated with peritoneal dissemination. In vitro experiments revealed that SEMA3C contributes to the process of peritoneal dissemination, which is composed of invasion, CSC properties, and colonization ability. We also found the relevance of SEMA3C expression levels and functions in phenotypes such as cell invasion, CSC properties, drug resistance, and tumor growth in the SEMA3C knockdown cells and overexpressing cells. Furthermore, we demonstrated that SEMA3C knockdown suppressed Akt/mTOR signal transduction via a decrease in SEMA3C/c-Met activation, leading to an increase in the efficacy of Gem in PDAC cells. Additionally, the combination of SEMA3C knockdown and GnP therapy facilitated tumor regression and impaired peritoneal dissemination in vivo. To our knowledge, this is the first demonstration of an association between SEMA3C, peritoneal dissemination, and CSC properties in PDAC (Fig. 7).

Axon guidance molecules, such as SEMAs, have been identified as mutated genes by whole exome sequencing and copy number analysis in early PDAC and are reported to be associated with pancreatic carcinogenesis through acinar-to-ductal metaplasia (ADM)[16]. Likewise, we previously described SEMAs as candidate downstream targets of Prrx1, a transcription factor associated with pancreatic development, regeneration with ADM, and PDAC progression [10]. Among class 3 SEMAs, SEMA 3 C/3D signaling is reportedly an evolutionarily conserved regulator of enteric nervous system development [17]. Foley et al. reported that annexin A2 (AnxA2) regulates the secretion of SEMA3D, promoting invasion and metastasis in PDAC [18]. Furthermore, Jurcak showed that the SEMA3D-Plexin D1 pathway mediates perineural invasion and metastasis in orthotopic murine PDAC models [19]. Notably, our clinical data demonstrated a significant correlation among SEMA3C expression, neural invasion, and peritoneal dissemination. These data suggest a novel avenue for elucidating the mechanistic relationship between invasive tumor dissemination and tumor-nerve signaling in PDAC.

According to the previous reports, IC_50_ values of Gem were analyzed to examine the Gem sensitivity in primary PDAC cell lines (BxPC-3, MIA PaCa-2, PANC-1) and the metastatic cell lines (AsPC-1, Capan-1, Hs766T). Hu et al. reported that the IC_50_ values of Gem were 13.683nM for BxPC-3, 7.723nM for MIA PaCa-2, 136.786nM for PANC-1, 568.354nM for AsPC-1, and 6.614nM for Capan-1, respectively [20]. We found that MIA PaCa-2 with lower SEMA3C expression showed higher sensitivity to Gem, while BxPC-3 and PANC-1 with higher SEMA3C expression showed the less sensitivity in primary PDAC cell lines. In the metastatic cell lines, AsPC-1 showing high IC_50_ values of Gem exhibited relatively low SEMA3C protein level in this study which data is controversial to our SEMA3C protein expressions by western blotting. Contrary to this in the metastatic PDAC cell lines, Espey et al. reported that IC_50_ of Gem for Hs766T (IC_50_: 2422µM) was more than 1,000-fold higher than that for AsPC-1 (IC_50_: 0.191µM), which is consistent with our results [21]. Although these IC_50_ values were investigated under the different conditions, these findings imply that there might be a correlation between intrinsic SEMA3C protein expression levels and resistance to Gem in PDAC cell lines.

In this study, we demonstrated that SEMA3C promotes peritoneal dissemination by regulating CSC properties and Gem resistance by accelerating Akt/mTOR signaling via phosphorylation of c-Met receptors in PDAC cells. The Akt/mTOR pathway is involved in Gem resistance through AnxA2 [22], and Akt/mTOR signaling is enhanced by the activation of c-Met [23], which plays a pivotal role in the maintenance of CSC properties in PDAC cells [14]. SEMA3C expression was positively correlated with an increase in the subpopulation of CD44^high^CD24^high^ in this study. However, the association between SEMA3C and AnxA2 should be clarified to elucidate the precise mechanism underlying Gem resistance.

In our preclinical study, SEMA3C knockdown enhanced the efficacy of GnP in reducing tumor growth and peritoneal carcinomatosis. In clinical applications, antisense oligonucleotides (ASOs), which influence RNA processing and modulate protein expression, could potentially inhibit SEMA3C [24, 25]. SEMA3C ASO treatment suppresses tumor growth in castration-resistant prostate cancer in vivo [26]. As IP chemotherapy is required to increase the concentration of drugs in peritoneal tumors, the intra-abdominal administration of amido-bridged nucleic acid-modified anti-SYT13 ASOs represents a promising strategy for treating peritoneal metastasis of gastric cancer [27]. Thus, SEMA3C ASOs are a candidate therapeutic agent for the peritoneal dissemination of PDAC.

A limitation of our study was that the function of SEMA3C in the immune TME was not investigated in a mouse model. SEMA3C promotes cancer cell survival by regulating autophagy and affecting the TME immune response [28]. Therefore, the functional roles of SEMA3C should be examined in the immune TME of PDAC.

## Conclusions

Our study demonstrated that combination therapy with SEMA3C inhibition and GnP impairs peritoneal dissemination by regulating CSC properties and overcoming Gem resistance by activating the Akt/mTOR pathway via c-Met. Future clinical studies are warranted to determine whether SEMA3C inhibition and Gem-based chemotherapy affect the control of peritoneal metastasis of PDAC.

### Electronic supplementary material

Below is the link to the electronic supplementary material.

Supplementary Material 1

Supplementary Material 2

Supplementary Material 3

Supplementary Material 4

Supplementary Material 5

Supplementary Material 6

Supplementary Material 7

## Data Availability

The datasets used and/or analyzed during the current study are available from the corresponding author on reasonable request.
