# Supplementary material for: Semaphorin 3 C enhances putative cancer stemness and accelerates peritoneal dissemination in pancreatic cancer
Source: Cancer Cell Int. 2023 Aug 3;23:155. doi: 10.1186/s12935-023-03008-3 (PMC10401755; doi:10.1186/s12935-023-03008-3)
Supplement: Supplementary file 1 — Supplementary Material 1 [file 12935_2023_3008_MOESM1_ESM.docx]

**Table S1 List of medium for each cell lines**

| **Human cell lines** | **Medium** |
| --- | --- |
| Human pancreatic duct epithelial (HPDE) | - keratinocyte serum-free medium (Gibco, Carlsbad, CA, USA) - bovine pituitary extract - epithermal growth factor |
| BxPC-3  AsPC-1 | - RPMI-1640 medium (Gibco) - 10% fetal bovine serum (FBS) |
| MIA PaCa-2  PANC-1  Hs766T | - Dulbecco’s modified Eagle medium (DMEM; Nacalai Tesque, Kyoto, Japan) - 10% FBS |
| Capan-2 | - McCoy’s 5A medium (Gibco) - 10% FBS |
| CFPAC-1 | - Iscove’s modified Dulbecco’s medium (Thermo Fisher Scientific, Waltham, MA) - 10% FBS |
| Capan-1 | - DMEM - 20% FBS |

| **Murine cell lines** | **Medium** |
| --- | --- |
| PKCY | - Dulbecco’s modified Eagle medium (DMEM; Nacalai Tesque, Kyoto, Japan) - 10% FBS |

**Table S2 List of primary and secondary antibodies**

| **Primary antibody** | **Sources** | **Dilution** |
| --- | --- | --- |
| Anti-SEMA3C polyclonal antibody | Abbexa, Cambridge, UK | IHC 1:500 |
| Anti-SEMA3C polyclonal antibody | Cloud-Clone Corp., Houston, TX, USA | WB 1:500 |
| Anti-mouse SEMA3C monoclonal antibody | R&D Systems, Minneapolis, MN, USA | WB 1:1000 |
| Anti-c-Met monoclonal antibody | Abcam, Cambridge, UK | WB 1:1000 |
| Anti-phospho-c-Met polyclonal antibody | Invitrogen, Carlsbad, CA, USA | WB 1:1000 |
| Anti-Akt monoclonal antibody | Cell Signaling Technology, Danvers, MA, USA | WB 1:1000 |
| Anti-phospho-Akt polyclonal antibody | Cell Signaling Technolog | WB 1:1000 |
| Anti-mTOR polyclonal antibody | Cell Signaling Technology | WB 1:1000 |
| Anti-phospho-mTOR polyclonal antibody | Cell Signaling Technology | WB 1:1000 |
| FITC anti-human CD44 antibody | BioLegend, CA, USA | FCS 1:10 |
| APC anti-human CD24 antibody | BioLegend | FCS 1:10 |
| PE anti-human CD133 antibody | BioLegend | FCS 1:10 |
| FITC anti-human c-Met antibody | BioLegend | FCS 1:10 |
| Anti-βactin monoclonal antibody | Cell Signaling Technology, MA, USA | WB 1:2000 |
| Anti-GAPDH polyclonal antibody | GeneTex Inc, CA, USA | WB 1:2000 |

| **Secondary antibody** | **Sources** | **Dilution** |
| --- | --- | --- |
| Envision^TM^+/ horseradish peroxidase (HRP) anti-rabbit antibody | DAKO, Santa Clara, CA, USA | IHC  No dilution |
| Anti-rabbit IgG HRP antibody | Santa Cruz Biotechnology, Santa Cruz, CA, USA | WB 1:2000 |
| Anti-rat IgG HRP antibody | Proteintech, Chicago, IL, USA | WB 1:2000 |

WB: western blotting, IHC: immunohistochemistry, FCS: flow cytometry standard

**Table S3 Primers sequences used for real-time PCR**

| Gene | Species | Sequence (5'-3') |
| --- | --- | --- |
| *SEMA3C* | Human | forward: 5’-CAAAGATCCCACACACGGCT-3’ |
|  |  | reverse: 5’- ACTTGGTCCTCTGATCTCCTCC-3’ |
| *SEMA3C* | Mouse | forward: 5’-CCTAATGACACTGGTGGACA-3’ reverse: 5’- CCTATGCGGACTATCAGAGG -3 |
| *β-actin* | Human | forward: 5’-TGGCACCCAGCACAATGAA-3’  reverse: 5’-CTAAGTCATAGTCCGCCTAG-3’ |
| *β-actin* | Mouse | forward: 5’-CATCCGTAAAGACCTCTATG-3’  reverse: 5’-ATGGAGCCACCGATCCACA-3’ |
