# Supplementary material for: Semaphorin 3 C enhances putative cancer stemness and accelerates peritoneal dissemination in pancreatic cancer
Source: Cancer Cell Int. 2023 Aug 3;23:155. doi: 10.1186/s12935-023-03008-3 (PMC10401755; doi:10.1186/s12935-023-03008-3)
Supplement: Supplementary file 2 — Supplementary Material 2 [file 12935_2023_3008_MOESM2_ESM.docx]

**Figure legends**

**Fig. S1** *SEMA3C* mRNA expression was evaluated by RT-PCR in BxPC-3 cells transfected with control siRNA, SEMA3C siRNA1, and SEMA3C siRNA2 (A), and MIA PaCa-2 cells transfected with blank and SEMA3C pcDNA (B).

**Fig. S2** Expression patterns of stem cell markers such as CD133 and c-Met were compared by flow cytometry in BxPC-3 cells transfected with control siRNA, SEMA3C siRNA1, and SEMA3C siRNA2 (A), and MIA PaCa-2 control and SEMA3C-overexpressing cells (B).

**Fig. S3** LDH cytotoxicity assay of GEM comparing MIA PaCa-2 cells transfected with control pcDNA blank and pcDNA SEMA3C.

**Fig. S4** (A) SEMA3C expression was evaluated in PKCY cells transduced with control and SEMA3C shRNA using western blot analysis. (B) The sphere formation rate in PKCY cells treated with control siRNA and SEMA3C shRNA. (C) Bubble formation after cell injection into the subcapsular region of the pancreas.

**Fig. S5** Change in the body weight of mice in each group after transplantation. Body weight is represented as the mean ± SEM.
