# Supplementary figures and images for: Semaphorin 3 C enhances putative cancer stemness and accelerates peritoneal dissemination in pancreatic cancer
Source: Cancer Cell Int. 2023 Aug 3;23:155. doi: 10.1186/s12935-023-03008-3 (PMC10401755; doi:10.1186/s12935-023-03008-3)

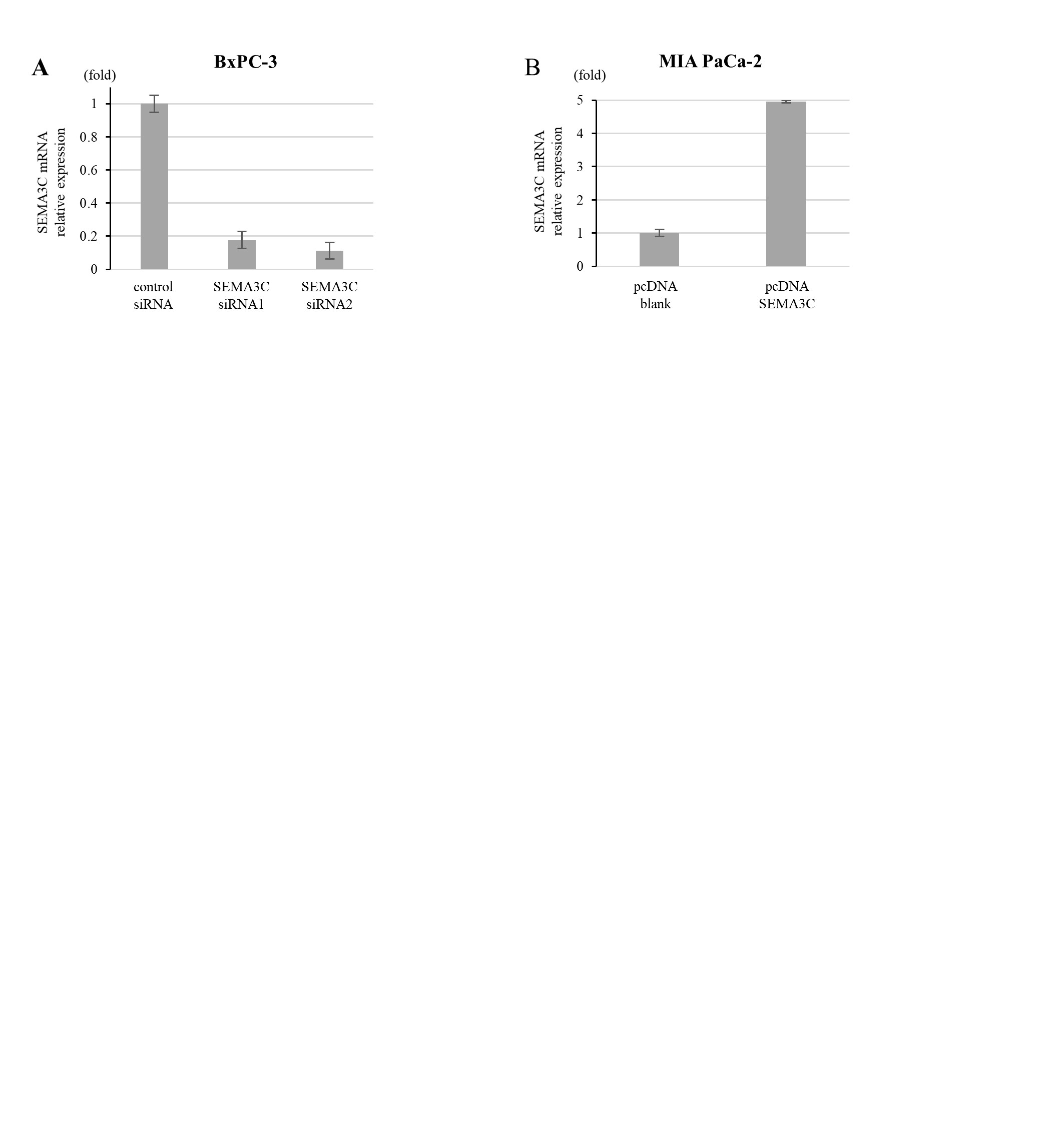

Supplement: Supplementary file 3 — Supplementary Material 3 [file 12935_2023_3008_MOESM3_ESM.jpg]

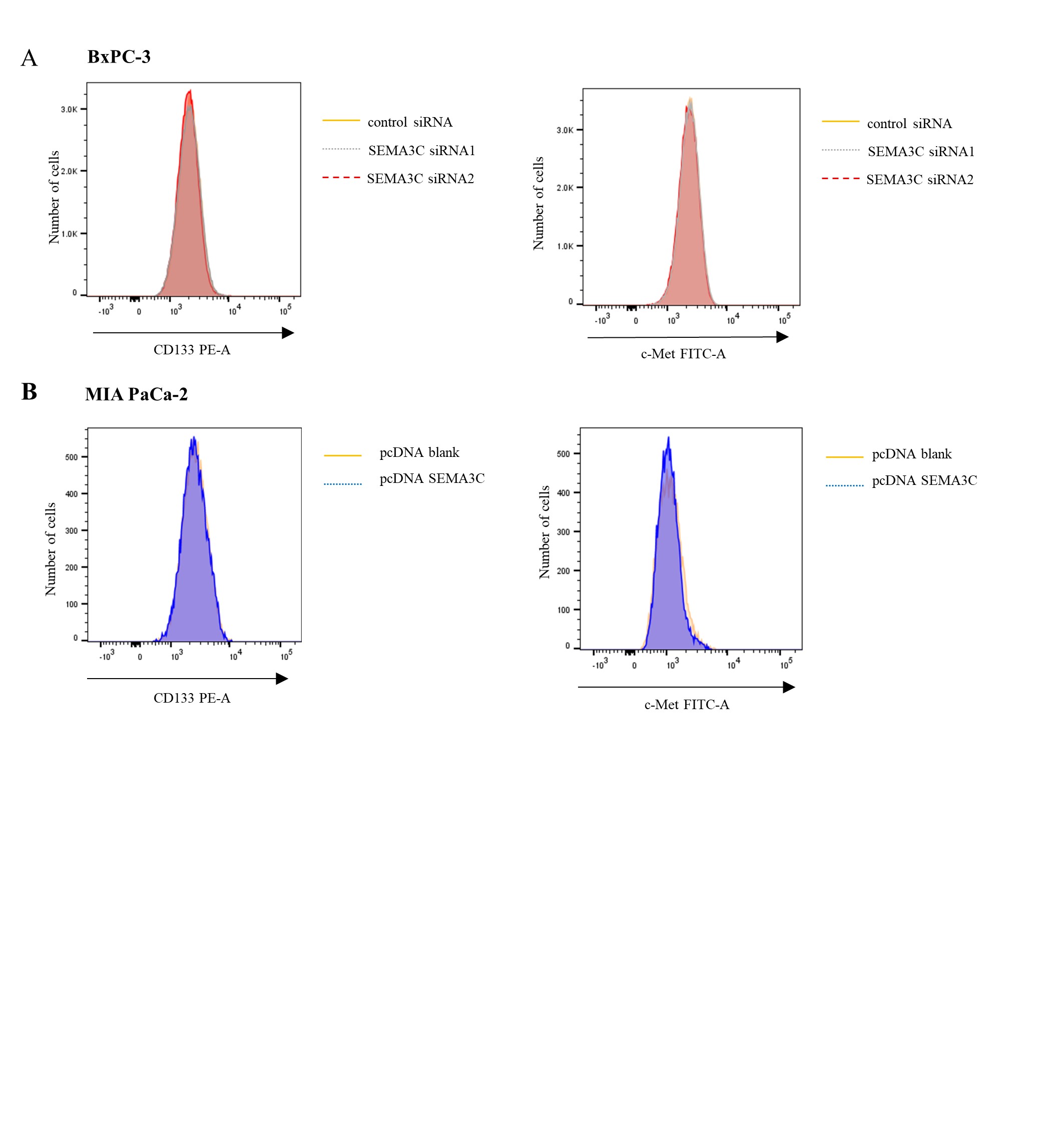

Supplement: Supplementary file 4 — Supplementary Material 4 [file 12935_2023_3008_MOESM4_ESM.jpg]

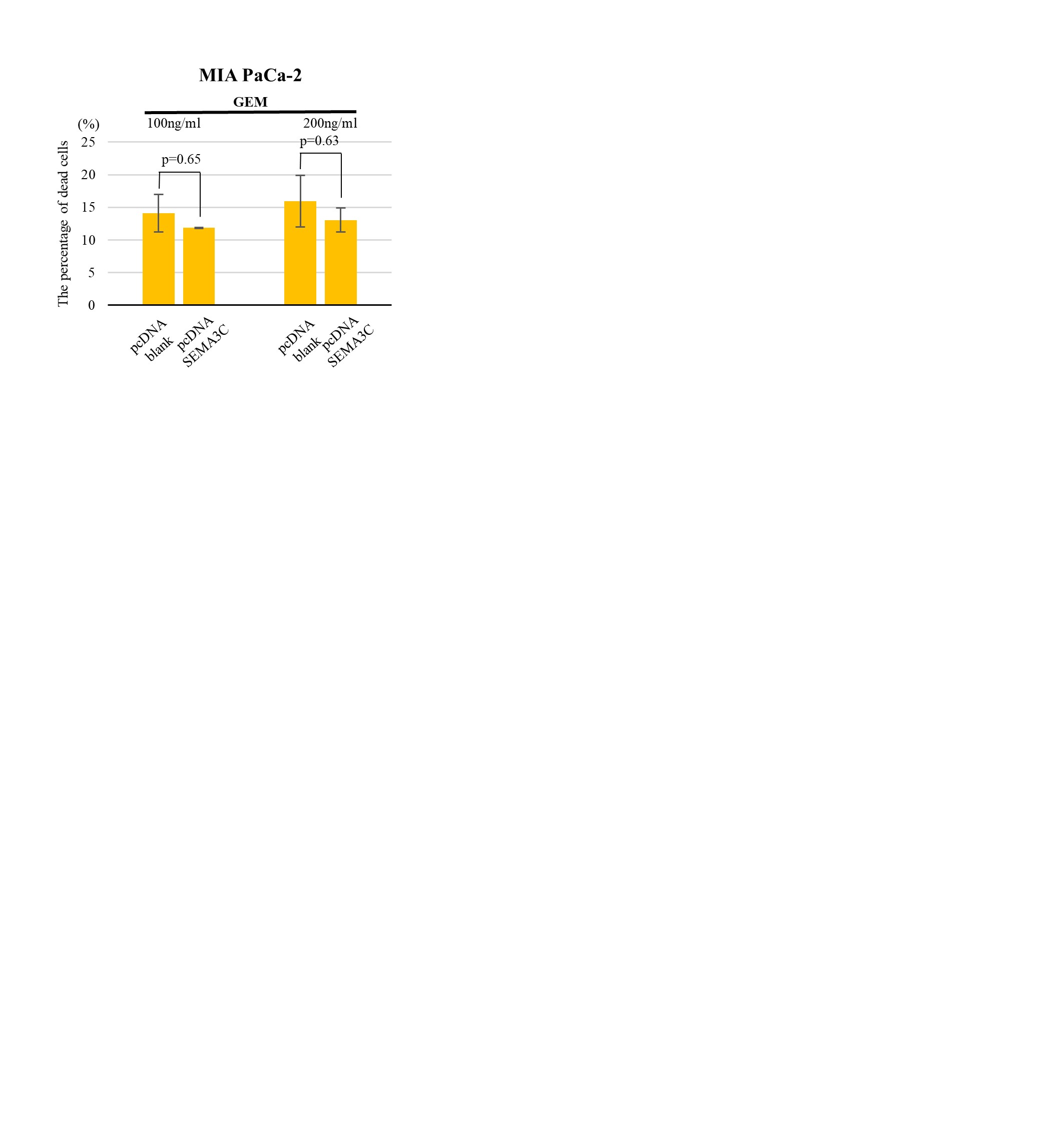

Supplement: Supplementary file 5 — Supplementary Material 5 [file 12935_2023_3008_MOESM5_ESM.jpg]

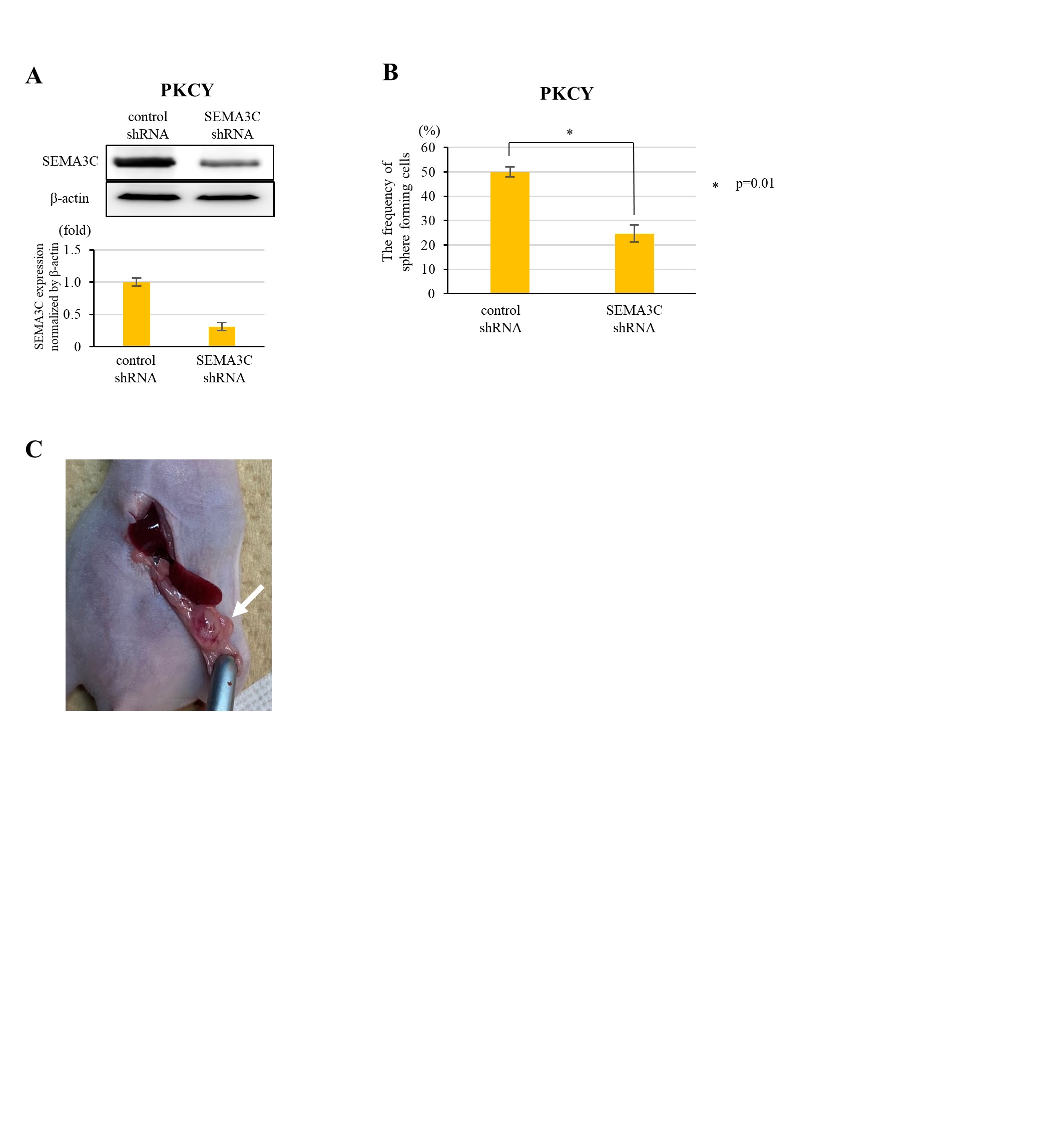

Supplement: Supplementary file 6 — Supplementary Material 6 [file 12935_2023_3008_MOESM6_ESM.jpg]

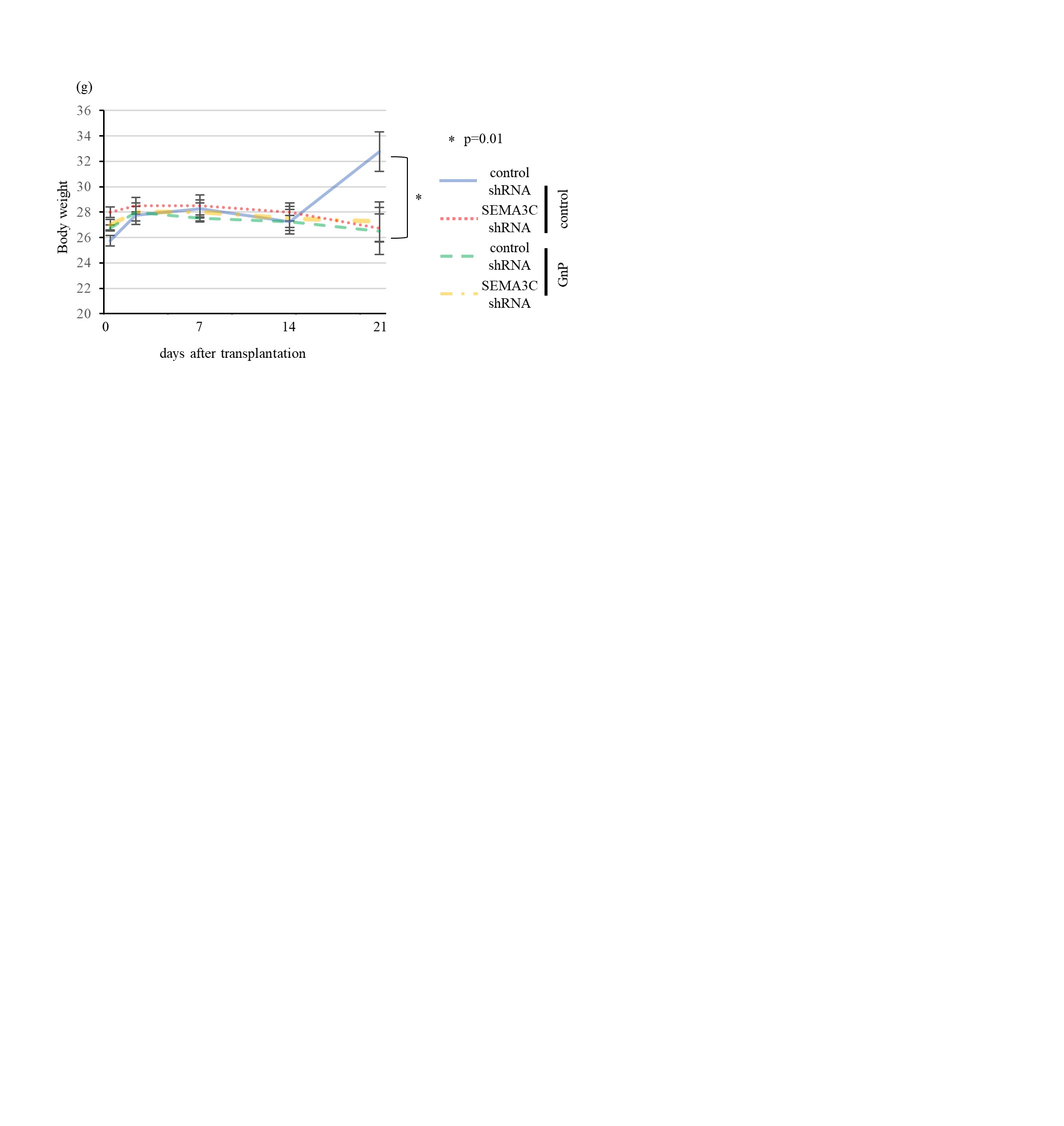

Supplement: Supplementary file 7 — Supplementary Material 7 [file 12935_2023_3008_MOESM7_ESM.jpg]
